# Plant nitrogen uptake and assimilation: regulation of cellular pH homeostasis

**DOI:** 10.1093/jxb/eraa150

**Published:** 2020-03-24

**Authors:** Huimin Feng, Xiaorong Fan, Anthony J Miller, Guohua Xu

**Affiliations:** 1 State Key Laboratory of Crop Genetics and Germplasm Enhancement, Nanjing, China; 2 MOA Key Laboratory of Plant Nutrition and Fertilization in Lower-Middle Reaches of the Yangtze River, Nanjing Agricultural University, Nanjing, China; 3 Metabolic Biology, John Innes Centre, Norwich Research Park, Norwich, UK; 4 Michigan State University, USA

**Keywords:** Ammonium, assimilation, ATPase, charge balance, cellular pH, homeostasis, nitrate, pump, transport, uptake

## Abstract

The enzymatic controlled metabolic processes in cells occur at their optimized pH ranges, therefore cellular pH homeostasis is fundamental for life. In plants, the nitrogen (N) source for uptake and assimilation, mainly in the forms of nitrate (NO_3_^–^) and ammonium (NH_4_^+^) quantitatively dominates the anion and cation equilibrium and the pH balance in cells. Here we review ionic and pH homeostasis in plant cells and regulation by N source from the rhizosphere to extra- and intracellular pH regulation for short- and long-distance N distribution and during N assimilation. In the process of N transport across membranes for uptake and compartmentation, both proton pumps and proton-coupled N transporters are essential, and their proton-binding sites may sense changes of apoplastic or intracellular pH. In addition, during N assimilation, carbon skeletons are required to synthesize amino acids, thus the combination of NO_3_^–^ or NH_4_^+^ transport and assimilation results in different net charge and numbers of protons in plant cells. Efficient maintenance of N-controlled cellular pH homeostasis may improve N uptake and use efficiency, as well as enhance the resistance to abiotic stresses.

## Teaser

Cellular pH homeostasis is regulated through the activities of N transporters and proton pumps affecting proton production or consumption during root acquisition, short and long-distance transport, and assimilation of N.

## Introduction

Nitrogen (N) is required for plants to complete their life cycles and is the most important nutrient acquired in greatest quantities by roots (Xu *et al.*, 2012; Oosterhuis *et al.*, 2014). NO_3_^–^ and NH_4_^+^ are the most prominent forms of inorganic N taken up by land plant species, and their root uptake rapidly causes primary effects on ionic and pH balance in plant cells. Cellular homeostasis of ions and pH is fundamental to basic cellular processes and is needed to maintain normal plant growth and development as well as responses to stresses (Bassil and Blumwald, 2014; Reguera *et al.*, 2015). In addition, pH varies within different intracellular compartments and the proton gradient is important for the viability of cells (Shen *et al.*, 2013).

Within plant cells, several compartments with different pH exist in parallel. The cytosol has pH values at 7.2–7.4 to ensure proper biochemical reactions (Schumacher, 2014), while the vacuole and apoplast maintain more acidic pH levels at 5.0–5.5 (Felle, 2001; Martinière *et al.*, 2013a; Shen *et al.*, 2013; Schumacher, 2014). Cytoplasmic pH (pHc) homeostasis is the result of a variety of processes. First, cytoplasmic chemical buffering components, such as bicarbonate, phosphate, and protein buffers, play important roles in stabilizing pHc (Kurkdjian and Guern, 1989). Secondly, the physical pH-stat, which is proton transport across membranes, contributes to pHc homeostasis (Felle 2001; Britto and Kronzucker, 2005). The maintenance of optimal pH in plant cells has to be tightly regulated and is established by different primary active H^+^ pumping complexes, such as the plasma membrane (PM) or P-type H^+^-ATPase (PM-ATPase), vacuolar H^+^-ATPase (V-ATPase), and the vacuolar H^+^-pyrophosphatase (V-PPase) (Schumacher, 2006; Gaxiola *et al.*, 2007; Marshansky and Futai, 2008). The P-type ATPases can be present in both the PM and vacuole (Li *et al.*, 2016). The physical pH-stat is also determined by transport of other ions to maintain the electrochemical balance, and H^+^-coupled ion transporters contribute to intracellular pH homeostasis (Gerendás and Schurr, 1999; Reguera *et al.*, 2015). Thirdly, a biochemical pH-stat participates in pHc regulation, including the metabolic processes of proton production or consumption, and organic acid production or degradation (Raven and Smith, 1976; Felle, 2001; Britto and Kronzucker, 2005). For example, the malate anion shuttle between the cytosol and vacuole is an important element of pHc regulation (Raven and Smith, 1976; Felle, 2001; Britto and Kronzucker, 2005). The primary root acquisition of NO_3_^–^ and/or NH_4_^+^ dominates anion and cation balance in plant cells, with uptake and vacuolar storage driven by PM-ATPases, V-ATPases, and V-PPases, while they consume energy and are essential components of cellular pH homeostasis providing a ‘physical pH-stat’ (Serrano, 1990; Barkla and Pantoja, 1996; Sze *et al.*, 1999; Martinoia *et al.*, 2000; Palmgren, 2001). In addition, the processes of NO_3_^–^ and NH_4_^+^ assimilation inside the cell are considered to consume or produce protons, contributing to ‘biochemical pH-stat’ (Britto and Kronzucker, 2005; Fan et al., 2016, 2017). In addition, NO_3_^–^ reduction leads to biochemical pH-stat by increasing malate and other organic acid anions (van Beusichem *et al.*, 1988; Lüttge *et al.*, 2000; Pasqualini *et al.*, 2001).

In this review, we summarize the general behaviours of N uptake, distribution, and assimilation inducing changes in plant cellular and rhizosphere pH. We discuss the regulatory mechanisms of the maintenance of cellular pH under altered N supplies in both physiology and molecular aspects.

## Regulation of pH by N acquisition: from cell to rhizosphere

In response to the uptake of varied N forms, plants change their ionic balance, cellular transmembrane electric potentials, and proton pumping activity, resulting in altered cellular and rhizosphere pH.

### N supply-controlled ionic and electronic balance in plants

Plant uptake of NH_4_^+^ or NO_3_^–^ accompanies the flux of other nutrient ions including K^+^, Cl^–^, and H^+^ for charge balance. It is well known that an antagonism or a cooperation between NH_4_^+^ or NO_3_^–^ and potassium (K^+^) arises from their charge and influence on the membrane potential, namely K^+^–NH_4_^+^ competition and K^+^–NO_3_^–^ cooperation (Li *et al.*, 2016; reviewed by Coskun *et al.*, 2017). NH_4_^+^ competes with low-affinity K^+^ uptake and accumulation (Wang *et al.*, 1996; Szczerba *et al.*, 2008; Hoopen et al., 2010; Chen *et al.*, 2015). The acquisition rates of cationic K^+^ and anionic NO_3_^–^ are often found to be positively correlated, probably due to improved charge balance or activation of the enzymes involved in NO_3_^–^ assimilation (Hagin *et al.*, 1990; Roosta and Schjoerring, 2008; Balkos *et al.*, 2010; Yang *et al.*, 2014; Xia *et al.*, 2015). NO_3_^–^ is transported from root to shoot with K^+^ as a counter ion in the xylem; thus, limited K^+^ supply can result in high accumulations of NO_3_^–^ in roots (Rufty *et al.*, 1981; Förster and Jeschke, 1993). Knockout of the nitrate transporter AtNPF7.3/NRT1.5 in Arabidopsis and OsNPF2.4 in rice not only decreased NO_3_^–^ loading to xylem sap, but also limited K^+^ content in the xylem (Lin *et al.*, 2008; Xia *et al.*, 2015; Li *et al.*, 2017), indicating the interaction of NO_3_^–^ and K^+^ in plant cells.

In the vacuole, the monovalent anions NO_3_^–^, malate, and Cl^–^ show an interaction; for example, the Cl^–^ concentration in leaves can be reduced by the NO_3_^–^ supply (Glass and Siddiqi, 1985; Guo *et al.*, 2017). Two maize nitrate transporters, ZmNPF6.4 and ZmNPF6.6, are permeable to both NO_3_^–^ and Cl^–^ (Wen *et al.*, 2017), indicating that the two anions could be facilitated by the similar transport systems in plants. There are also chloride-specific MATE transporters in the vacuolar membrane (Zhang *et al.*, 2017). Diurnal changes in vacuolar malate have been observed to compensate for NO_3_^–^ and K^+^ fluctuations (Niedziela *et al.*, 1993).

### Instant response of cellular membrane potential and pH

The cell membrane potential (∆Ψ, negative inside the cell compared with outside the cell) can be affected by fluxes of charged ions across the PM. An immediate physiological response of root cells to NH_4_^+^ and NO_3_^–^ exposure is a transient change of ∆Ψ, which is caused by NH_4_^+^ and NO_3_^–^ inﬂux carrying H^+^ into the cell and compensated by activation of the PM H^+^-ATPase to repolarize and maintain ∆Ψ (Ullrich and Novacky, 1990; Wang *et al.*, 1994; Liu *et al.*, 2017). However, the initial membrane depolarization was not commensurate with the increased influx of NH_3_/NH_4_^+^ (p*K*a 9.25) at pH 6.25 in the medium in roots of barley, suggesting that the increased transport of electroneutral NH_3_ dominates uptake (Coskun *et al.*, 2013). NO_3_^–^ is co-transported with H^+^ through a symporter into cells, and the stoichiometry of NO_3_^–^ and H^+^ is ~2 (Glass *et al.*, 1992; Miller and Smith, 1996). Root NO_3_^–^ acquisition commonly leads to ∆Ψ depolarization of the cells suggesting an H^+^ stoichiometry >1 (Meharg and Blatt, 1995; Mistrik and Ullrich, 1996; Britto and Kronzucker, 2005).

It is controversial whether such transport mechanisms would lead to longer term cytosol alkalinization by NH_4_^+^/NH_3_ uptake or acidification by NO_3_^–^ uptake, but at least in the initial period after the addition of NH_4_^+^ or NO_3_^–^ some pH changes are generally accepted. For NO_3_^–^ uptake, only small changes in cytoplasmic pH occurred in roots of maize seedlings growing in nutrient solutions at different pH and supplemented with normal NO_3_^–^ (5 mM) (Gerendás *et al.*, 1990). It is proposed that these results are attributed to the presence of tight regulatory mechanisms for intracellular pH. An important component of NH_4_^+^/NH_3_ or NO_3_^–^ uptake in plants is the assimilatory consumption of these ions. An initial NO_3_^–^-induced cytosolic acidification was measured in *Limnobium stoloniferum* root hairs (Raven, 1985, 1986; Ullrich and Novacky, 1990). NO_3_^–^ assimilation, which is a proton-consuming process, might cause an increase of cytoplasmic pH and thus partially compensate for H^+^ influx coupled with NO_3_^–^ uptake. In maize roots, the inhibition of NO_3_^–^ assimilation using tungstate, an inhibitor of NO_3_^–^ reductase activity, resulted in acidification of the cytosol (Espen *et al.*, 2004). Another regulatory mechanism to prevent NO_3_^–^ uptake generating acidification of the cytoplasm is an increase in PM-ATPase activity. Decreased cytoplasmic pH is a signal triggering the PM-ATPase to pump H^+^ out of the cytosol (Espen *et al.*, 2004) and hyperpolarize the PM ∆Ψ (Glass *et al.*, 1992; McClure et al., 1990*a*, *b*). In contrast to NO_3_^–^, the effect of NH_4_^+^ uptake on intracellular pH is dependent on external medium pH (Gerendás *et al.*, 1990; Kosegarten *et al.*, 1997; Gerendás and Ratcliffe, 2000). Maize root tip intracellular pH showed no change at external pH 6, but decreased at pH 4 and increased at pH 8 with 5 mM NH_4_^+^ supply (Gerendás *et al.*, 1990). At high external pH, the NH_4_^+^/NH_3_ equilibrium shifts in favour of the NH_3_ molecule that readily permeates the PM through aquaporins (Kleiner, 1981; Macfarlane and Smith, 1982; Coskun *et al.*, 2013). At external pH 9, both the cytosol and vacuole were alkalinized in 1 h with NH_4_^+^ supply from 5 mM to 20 mM (Gerendás and Ratcliffe, 2000). Both NH_4_^+^ transport and assimilation were assumed to contribute to the alkalinization of cytosolic pH (Kosegarten *et al.*, 1997). In the external pH range from 5 to 7, the cytoplasmic buffer capacity may be able to balance the NH_4_^+^-elicited pH changes (Kosegarten *et al.*, 1997).

Some caution is needed when evaluating the influence of other accompanying cations (e.g. K^+^, Mg^2+^, or Ca^2+^) and anions (e.g. Cl^–^) on the alteration of cellular pH grown with NO_3_^–^ and NH_4_^+^ supply. For example, increased H^+^/K^+^ antiport at the PM under high K^+^ supply may compensate for the NO_3_^–^ uptake-induced cytosolic acidification via 2H^+^/NO_3_^–^ symport (Kurkdjian and Guern, 1989; Ullrich and Novacky, 1990; Guern *et al.*, 1991; Briskin and Hanson, 1992; Sacchi and Cocucci, 1992; Nocito *et al.*, 2002).

### Activity of ATPase and PPase in response to alternative supplies of N

The activity of membrane ATPases, PPases, and H^+^-coupled transporters establishes and can regulate cytoplasmic pH homeostasis. The PM H^+^-ATPase plays an important physiological role in maintaining the plasma membrane electrical potential difference and generating a transmembrane H^+^ chemical gradient (∆H; acidic on the outside) during the uptake of nutrients (Palmgren, 2001; Falhof *et al.*, 2016). For example, it was found that adding PM H^+^-ATPase inhibitors dramatically decreased root NO_3_^–^ uptake (McClure *et al.*, 1990*b*), and eliminated the NH_4_^+^ uptake-generated depolarization of ∆Ψ (Wang *et al.*, 1994). In early adjustment to N uptake, the PM H^+^-ATPase plays an important role in maintaining cytosolic pH homeostasis. When compared with CaSO_4_ solution, (NH_4_)_2_SO_4_ induced the PM H^+^-ATPase activity in roots of barley seedlings (Yamashita *et al.*, 1995). Similarly, Ca(NO_3_)_2_ treatment also induced a significantly higher transcription of PM-ATPase genes after a 3 h exposure and a significantly higher protein concentration and activity after a 6 h exposure (Santi *et al.*, 2003). Interestingly, PM H^+^-ATPase activity including both hydrolytic and H^+^-pumping activity and its related gene expression showed no difference in rice plants grown in 2.5 mM NH_4_^+^ or NO_3_^–^ solution when the solution was buffered at the same pH (Zhu *et al.*, 2009).

NO_3_^–^ transport into the vacuole from the cytosol is mediated by an H^+^/NO_3_^–^ antiport mechanism, which is driven by P- and V-type ATPases and V-PPase activity (Granstedt and Huffaker, 1982; Blumwald and Poole, 1985; Schumaker and Sze, 1987; Glass *et al.*, 1992; Miller and Smith, 1992; Krebs *et al.*, 2010). High concentrations of NO_3_^–^ could inhibit V-ATPase activity in isolated vacuoles (Blumwald and Poole, 1985). Inhibiting the activity of V-ATPase or V-PPase or knockout of their encoding genes significantly decreased NO_3_^–^ storage and influx into vacuoles of *Brassica napus* plants (Han *et al.*, 2016).

### Factors dominating N supply effects on rhizosphere pH

Soil alkalinity above pH 8.0 or acidity below pH 5.5 limits plant growth and development (Schubert *et al.*, 1990; Koyama *et al.*, 2001; Cha-Um *et al.*, 2009; Patil *et al.*, 2012). Uptake of NH_4_^+^ or NO_3_^–^ (i.e. transport and assimilation) results in rapid acidification or alkalinization of the apoplast (Geilfus, 2017) and rhizosphere (Taylor and Bloom 1998; Kosegarten *et al.*, 1997; Gerendás and Ratcliffe, 2000; Ruan *et al.*, 2000; Hinsinger *et al.*, 2003). It has been shown that decreasing external pH to acidic levels can up-regulate the expression of 20–41% of the NH_4_^+^-responsive genes in *Arabidopsis thaliana*, suggesting that apoplastic acidification is a component of NH_4_^+^-induced stress (Patterson *et al.*, 2010).

**Fig. 1.**
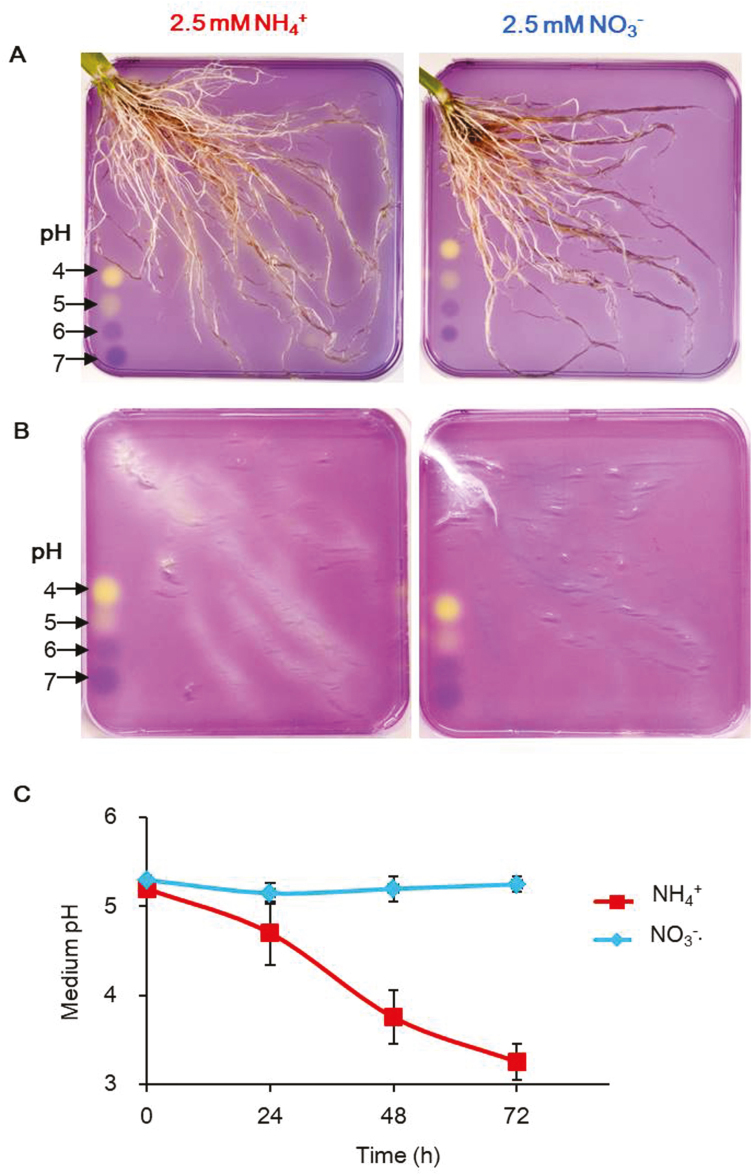
Rhizosphere pH regulated by uptake of NH_4_^+^ and NO_3_^–^ in rice roots. (A) The rhizosphere pH of rice roots shown with a colour pH indicator. (B) Agar profile showing rhizosphere pH after removing the roots. Rice seedlings (*Oryza sativa* L ssp. *japonica*, Nipponbare) were grown in full nutrient solution containing 1.25 mM NH_4_NO_3_ for 4 weeks and then transferred to 2.5 mM NH_4_^+^ or 2.5 mM NO_3_^–^ for 72 h. After 72 h N treatment, the plant root was washed by dipping in 0.2 mM CaSO_4_ for 1 min before placement on the agar. An intact plant was placed on agar (0.9 g l^–1^, containing the pH indicator (0.03 g l^–1^ bromocresol purple). The initial pH was 5.2–5.3 from 11.00 h to 11.30 h, roots were kept in darkness covered with a moist paper tissue and under a 0.5×12×12 cm^3^ Plexiglas plate, and the picture was taken after 2–4 h in contact with the pH indicator agar. (C) pH of the hydroponic growth medium during 2.5 mM NH_4_^+^ or 2.5 mM NO_3_^–^ solution after 24, 48, and 72 h. The initial pH was 5.2–5.3.

The N supply factors causing changes in rhizosphere or apoplastic pH include N concentrations and forms, balance of N with other major nutrients, and plant species. (i) High NH_4_^+^ supply induced rhizosphere acidification and high NO_3_^–^ induced alkalinization (Marschner and Römheld, 1983; Römheld, 1986; Hinsinger *et al.*, 2003) controlled by the processes of N transport (see ‘Extra- and intracellular pH regulation at short- and long-distance N distribution’) and assimilation (see ‘Cellular pH homeostasis during N assimilation’). (ii) For charge balance, NO_3_^–^ may increase, while NH_4_^+^ decreases, cation uptake by root cells. The imbalanced uptake of cations and anions triggers release of H^+^ or OH^–^ (or HCO_3_^–^) into the apoplast, resulting in opposing pH changes in the rhizosphere (Haynes, 1990; Marschner, 1995; Hinsinger *et al.*, 2003). (iii) The extent of the N supply-induced pH change in the rhizosphere or apoplast is also dependent on plant species. For example, the rhizosphere of lentils and chickpea could be acidified even at relatively high NO_3_^–^ supply (Römheld, 1986). The effects of N supply on rhizosphere pH can be simply shown using pH indicators in agar (see Fig. 1 for rice).

## Extra- and intracellular pH regulation at short- and long-distance N distribution

**Fig. 2.**
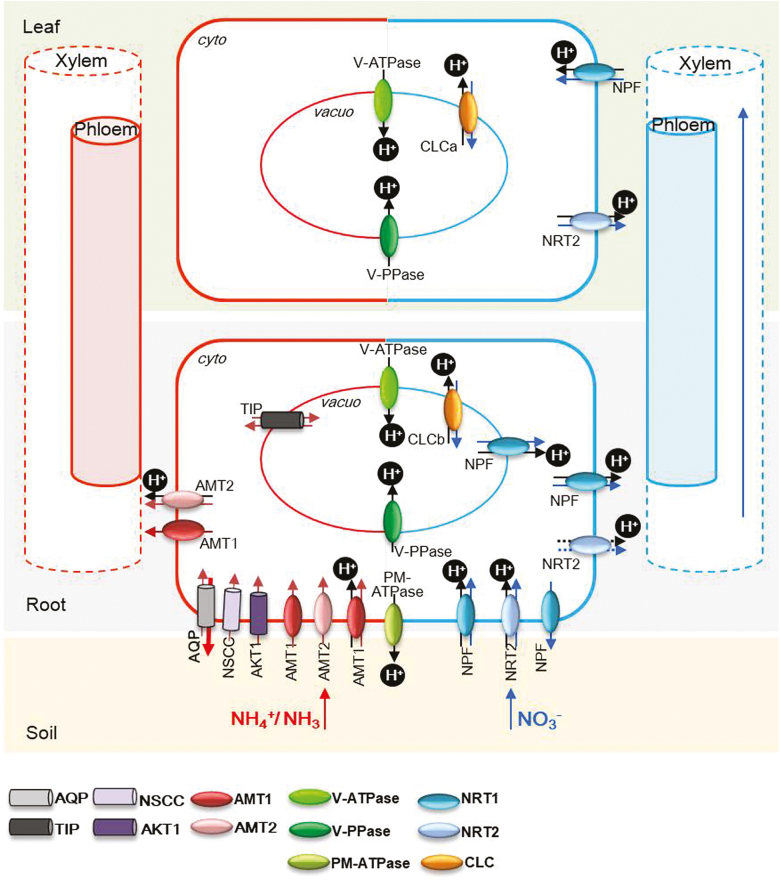
Protons are involved in NH_4_^+^ and NO_3_^–^ fluxes. Different transporters or channels for the fluxes of NH_4_^+^ (red arrow), NO_3_^–^ (blue arrow), and H^+^ (black arrow). Potassium channels (AKT1), non-selective cation channels (NSCC), and aquaporins (AQP, TIP) are NH_4_^+^/NH_3_ channels (Hachiya and Sakakibara, 2017; Liu and von Wirén, 2017). AMT1 is an NH_4_^+^ transporter functioning as an NH_4_^+^ or NH_3_ channel, NH_4_^+^ uniporter, or H^+^/NH_4_^+^ antiporter (Giehl *et al.*, 2017; Duan *et al.*, 2018; reviewed by Tegeder and Masclaux-Daubresse, 2018). NPF and NRT2 are plasma membrane (PM) or tonoplast NO_3_^–^ transporters functioning as an H^+^/ NO_3_^–^ symporter or an NO_3_^–^ excretion transporter (reviewed by Fan *et al.*, 2017; Wang *et al.*, 2018). CLCa and CLCb are tonoplast-localized chloride transporters functioning as H^+^/NO_3_^–^ antiporters (reviewed by Zifarelli and Pusch, 2010). Intracellular pH maintenance is also established by different primary active H^+^ pumping complexes, such as the PM H^+^-ATPase (PM-ATPase), the vacuolar H^+^-ATPase (V-ATPase), and V-PPase (reviewed by Gaxiola *et al.*, 2007). Cyto, cytosol. Vacuo, vacuole.

A variety of root and shoot NH_4_^+^ and NO_3_^–^ transporters may be involved in cellular pH homeostasis through the processes of H^+^ production or consumption within cellular compartments (Fig. 2). Cellular pH homeostasis is also dependent on the activity of the proton pumps, the PM-ATPase, V-ATPase, and V-PPase (Fig. 2). NH_4_^+^ transport is controlled by NH_4_^+^ transporters (AMTs) and non-saturable low-affinity uptake systems (i.e. aquaporins TIPs or cation channels) in plants. NO_3_^–^ transport is mediated by the NO_3_^–^ Transporter (NRT1 and NRT2) family, and the NRT1 family is renamed the NO_3_^–^ Transporter1/Peptide Transporter Family (NPF) (Léran *et al.*, 2013). The Chloride Channel (CLC) family also function as anion/proton exchangers or anion channels (De Angeli *et al.*, 2006), mediating NO_3_^–^ transport at the vacuole or in endomembrane vesicles (Zifarelli and Pusch, 2010).

For inorganic N transporters in plants, readers are also referred to previously published reviews (Léran *et al.*, 2013; Fan *et al.*, 2017; Tegeder and Masclaux-Daubresse, 2018; Wang *et al.*, 2018). Here we focus on the plant NH_4_^+^ and NO_3_^–^ transporters which are involved in maintaining pH balance both *in vitro* and *in vivo*.

### H^+^/NO_3_^–^ symporters are involved in regulation of cellular pH and ion homeostasis

Both NO_3_^–^ and NH_4_^+^ can be imported into root cells by H^+^-coupled symporters across the PM through energetically uphill processes. Most members of the nitrate transporter families NPF/NRT1 and NRT2 showed characteristics of pH-dependent NO_3_^–^ transport when expressed in *Xenopus laevis* oocytes. After injection of the *NPF/NRT1* and *NRT2* genes, the oocytes showed NO_3_^–^-elicited inward current and the pH dependency (i.e. NO_3_^–^-induced current is larger at pH 5.5 than at pH 7.4) that is associated with a H^+^-symport mechanism (Søgaard *et al.*, 2009; Ortiz-Ramirez *et al.*, 2011; Fan *et al.*, 2017; Wang *et al.*, 2018). Many results indicate that the NPFs function as H^+^/NO_3_^–^ co-transporters, which mediate the influx with the H^+^/NO_3_^–^ ratio being greater than one (Zhou *et al.*, 1998; Lin *et al.*, 2008). AtNPF6.3/NRT1.1/CHL1 is one of the exceptions, which is identified as both a pH-dependent importer (Tsay *et al.*, 1993; Liu *et al.*, 1999; Wang *et al.*, 2018) and a pH-independent exporter (Léran *et al.*, 2013). AtNPF6.3/NRT1.1/CHL1 knockout (point mutation of P492L, *chl1-9*) led to impaired H^+^ tolerance and the disappearance of alkalinization in NO_3_^–^-sufficient growth medium (Fang *et al.*, 2016), indicating that NRT1.1-mediated NO_3_^–^ uptake contributes to plant H^+^ tolerance by alkalinization of the rhizosphere. However, knockout of other nitrate transporters such as AtNPF4.6/ATI1/NRT1.2, AtNRT2.1, AtNRT2.2, and AtNRT2.4 did not alter the plant H^+^ tolerance (Fang *et al.*, 2016). Since NRT1.1 may contribute to root NO_3_^–^ uptake by 70–80% (Huang *et al.*, 1996; Wang *et al.*, 1998; Orsel *et al.*, 2004; Krouk *et al.*, 2010; Kiba *et al.*, 2012), it is possible that the activity of NRT1.1 masked the effect of other H^+^-coupled NO_3_^–^ transport in the tolerance to rhizosphere acidity. Furthermore, the mechanism of H^+^ movement via water molecules in the peptide-binding site for some members of the NRT1/NPF/POT family of secondary active transporters was suggested to provide a mechanism enabling the proteins to transport many diverse substrates (Parker *et al.*, 2017). Effectively, this mechanism separates substrate recognition from H^+^ translocation in this family of transporters.

Two members of the plant AMT family, common bean AMT1;1 and wheat AMT1;1, are characterized as H^+^-coupled importers. Expression of common bean *PvAMT1;1* in oocytes led to NH_4_^+^-elicited inward currents and cytosolic acidification, indicating that it functions as an H^+^/NH_4_^+^ symporter in a 1:1 ratio (Ortiz-Ramirez *et al.*, 2011). The activity of PvAMT1;1 was enhanced by low extracellular pH (pH 5.5), and this was demonstrated by changes in the reversal potential and by increased cytoplasm acidification measured with pH-selective microelectrodes (Ortiz-Ramirez *et al.*, 2011). However, there was no direct evidence to show whether PvAMT1;1 was related to H^+^ exchange in both the cytosol and rhizosphere *in vivo*.

Currently, it is not clear if xylem pH is regulated by H^+^/NO_3_^–^ co-transport. A PM NO_3_^–^ transporter, AtNPF7.3/NRT1.5, which is abundantly expressed in the pericycle or xylem parenchyma cells, mediates both pH-dependent NO_3_^–^ influx and efflux in oocytes, and release of NO_3_^–^ from the Arabidopsis root pericycle (Lin *et al.*, 2008). These authors proposed that there is a potential link between xylem pH and root-to-shoot NO_3_^–^ transport. However, AtNPF7.3/NRT1.5 is also identified as a H^+^-coupled H^+^/K^+^ antiporter in *Xenopus* oocytes, and functions in facilitating K^+^ loading into the xylem (Li *et al.*, 2017). Thus, it is unclear whether the long-distance transport of NO_3_^–^ and/or K^+^ contributed by NPF/NRT1s such as AtNPF7.3/NRT1.5 can alter pH in the xylem.

The NRT2s are another important family of NO_3_^–^ transporters, mediating uptake from the soil and transport to leaf cells and developing seeds (Xu *et al.*, 2012; Fan *et al.*, 2017). One of the first members of this family to be functionally characterized in oocytes was suggested not only to be an H^+^-coupled NO_3_^–^ symporter, but also to operate in an NO_3_^–^ transport mode uncoupled to H^+^ movement (Zhou *et al.*, 2000). This alternative mechanism may be beneficial when external NO_3_^–^ is very abundant, avoiding the pH problems that might be associated with H^+^ influx and cytosolic acidification. Some of the NRT2 transporters require a partner protein (NAR2) for function (Orsel *et al.*, 2006; Feng *et al.*, 2011; Yan *et al.*, 2011). In both Arabidopsis and rice, it has been shown that NAR2 is required for the targeting of the NRT2 protein from internal membrane vesicles to the PM (Wirth *et al.*, 2007; Liu *et al.*, 2014). The accumulation of the NRT2 transporter protein may provide a mechanism for altering the pH of these endomembrane vesicles.

 In the rice genome, the *OsNRT2.3* gene encodes two members of a H^+^-coupled nitrate transporter family, OsNRT2.3a and OsNRT2.3b (Feng *et al.*, 2011; Yan *et al.*, 2011). OsNRT2.3a is located in root stellar cells and plays an important role in distribution of NO_3_^–^ from root to shoot (Tang *et al.*, 2012), while OsNRT2.3b is expressed in phloem and contributed to phloem pH and ion homeostasis (Fan *et al.*, 2016). OsNRT2.3b expression in oocytes elicited a depolarized membrane potential and cytosolic acidification in response to NO_3_^–^ supply (Fan *et al.*, 2016). Notably, OsNRT2.3b functions only at a slightly alkaline cytosolic pH, and a pH-sensitive motif of OsNRT2.3b facing the cytosolic side determines its activity to acquire NO_3_^–^ from the external medium (Fan *et al.*, 2016). In rice, *OsNRT2.3b* overexpression decreased the phloem sap pH from 8 to 7.1 under NO_3_^–^ supply, and from 7.4 to 6.8 under NH_4_^+^ supply, resulting in significantly increased grain yield and nitrogen use efficiency (NUE) at different N levels in field conditions (Fan *et al.*, 2016). The sensing of cytosolic pH by OsNRT2.3b provides an explanation for plant adaptation to changes in the form of N supply. This finding highlights the important link between N transport, pH regulation, and NUE.

### NO_3_^–^ excretion transporters may be involved in cellular pH regulation

In contrast to NO_3_^–^ influx, NO_3_^–^ efflux from root cells is energetically a downhill process which is also dependent on the activity of the PM H^+^-ATPase pump. It was shown that in isolated root PMs, NO_3_^–^ efflux is tightly coupled to H^+^ excretion by the H^+^-ATPase, and that both activities of NO_3_^–^ efflux and H^+^ excretion share similar acidic optimum pH at the cytosolic face of the PM (Vara and Serrano, 1982; De Michelis and Spanswick, 1986; Grouzis *et al.*, 1997; Pouliquin *et al.*, 2000). It has been shown that the Nitrate Excretion Transporter AtNPF2.7/NAXT1 mediates passive NO_3_^–^ efflux across the isolated PM of plant root cells in acidic medium *in vitro* (Segonzac *et al.*, 2007), suggesting that the NO_3_^–^ excretion transporter can mediate both NO_3_^–^ and H^+^ efflux in combination with PM proton pumps, thus re-balancing the acidification of cytosol to some extent.

### Intracellular H^+^/NO_3_^–^ antiporters involved in pH regulation of cellular organelles

NO_3_^–^ can be stored in, and remobilized from, vacuoles. NO_3_^–^ transport into vacuoles is mediated by an H^+^/NO_3_^–^ antiporter, and the H^+^/NO_3_^–^ symport systems also serve in NO_3_^–^ efflux from the vacuole to the cytosol, which are energized by V-ATPase pumping H^+^ to vacuoles (De Angeli *et al.*, 2006). Arabidopsis *AtCLCa* is expressed in leaf mesophyll cells; disruption of *AtCLCa* led to an ~50% decrease of vacuolar NO_3_^–^, suggesting an important role for *AtCLCa* in NO_3_^–^ accumulation (Geelen *et al.*, 2000; De Angeli *et al.*, 2006). Measurements using the patch-clamp technique in the whole-vacuole configuration showed that AtCLCa behaves as a 1NO_3_^–^/2H^+^ exchanger, which transports NO_3_^–^ from the cytosol to the vacuolar lumen (De Angeli *et al.*, 2006). *AtCLCa* expression in oocytes indeed induced intracellular alkalinization at both pH 5.5 and pH 7.5 when oocytes were pulsed to positive voltages (Bergsdorf *et al.*, 2009). *In vitro*, although transport processes such as the H^+^/NO_3_^–^ exchanger AtCLCa play a role in alkalinization of the vacuole or acidifying the cytosol, the active accumulation of H^+^ in the vacuole is also accomplished by P- and V-type ATPases, which function as ‘proton pumps’. There are two ATP-binding sites, at His620 and Asp750 in the C-terminus CBS domain of AtCLCa. Adding micromolar concentrations of ATP could inhibit AtCLCa activity in isolated *A. thaliana* vacuoles, resulting in a decrease of NO_3_^–^ influx by up to 60% (De Angeli *et al.*, 2009). It is possible that the V-ATPases can work together with the CLC antiporter in the tonoplast to balance cytoplasmic pH during the process of vacuolar NO_3_^–^ accumulation.

Currently, it is not known if there are nitrate transporters involved in NO_3_^–^ flux and pH homeostasis in other cellular organelles. As members of all the NO_3_^–^ transporter families (NRT1, NRT2, and CLCs) can be located in endomembrane systems, they may have important roles in the generation of compartmental pH gradients within the cell.

### pH regulatory sites in N transporters

The activity of many NO_3_^–^ transporters is affected by pH; however, the regulatory mechanism is not clear. Interestingly, many plant N transporters including H^+^/NO_3_^–^ symporters, H^+^/NO_3_^–^ antiporters, and H^+^/NH_4_^+^ symporters contain putative pH-sensing sites (Table 1), indicating that these transporters may sense either external (i.e. apoplast) or internal pH.

**Table 1.** pH-sensing sites in plant ammonium and nitrate transporters

Transporter	Transport mode	pH sensing site	Localization	References
AtNPF6.3/NRT1.1/CHL1	2 H^+^/1 NO_3_^–^ symport	ExxER (E41, E44), His365 (H365)	PM	Sun *et al.* (2014); Parker and Newstead (2014)
PvAMT1;1	1 H^+^/1 NH_4_^+^ symport	His211 (H211)	PM	Ortiz-Ramirez *et al.* (2011)
OsNRT2.3b	2 H^+^/1 NO_3_^–^ symport	His167 (H167)	PM	Fan *et al.* (2016)
AtCLCa	1 H^+^/2 NO_3_^–^ antiport	Glu203 (E203), Glu270 (E270)	Tonoplast	Bergsdorf *et al.* (2009); Miller and Nguitragool (2009)

At, Arabidopsis; Pv, common bean; Os, rice. PM, plasma membrane; E, glutamate; H, histidine.

Both the ExxER motif and histidine residues are essential for H^+^ binding in plant NPFs (Jorgensen *et al.*, 2015; Longo *et al.*, 2018). Removal of charged residues in the ExxER motif of AtNPF6.3/NRT1.1 abolished both H^+^ binding and NO_3_^–^ transport activity (Sun *et al.* 2014). The stoichiometry of H^+^/NO_3_^–^ transport through AtNPF6.3/NRT1.1 is at least 2H^+^:1NO_3_^–^, and it was proposed that the ExxER motif in TM1 binds one H^+^, leaving His356 on TM7 to bind another H^+^ and NO_3_^–^ (Parker and Newstead, 2014).

It is well known that histidine residues are important H^+^-binding amino acids involved in the regulation or activity of pH-dependent transporters in *Escherichia coli*, yeast, mammals, and plants, because they can ionize within the physiological pH range (Wiebe *et al.*, 2001; Ortiz-Ramirez *et al.*, 2011). PvAMT1;1 is an NH_4_^+^ transporter of common bean, for which the mutation of its conserved His211 to glutamic acid (H211E) results in altering the transport mechanism to be pH independent, with its affinity for NH_4_^+^ decreasing while increasing the transport capacity (Ortiz-Ramirez *et al.*, 2011). Exposure of PvAMT1;1 H211E-expressing oocytes to NH_4_^+^ did not affect the cytoplasmic pH but caused depolarization of the membrane potential at both pH 5.5 and pH 7.0 (Ortiz-Ramirez *et al.*, 2011). For a rice NO_3_^–^ transporter, OsNRT2.3b, His167 (H167) was located on the cytoplasmic side and has been confirmed to play a critical role in sensing cytosolic pH (Fan *et al.*, 2016). The H167R mutation does not fully eliminate the basic activity of OsNRT2.3b as a H^+^-coupled NO_3_^–^ transporter but results in the loss of cytosolic pH sensing (Fan *et al.*, 2016).

 Certain gating glutamate residues of some channel proteins may be involved in sensing cellular pH. Mutation of AtCLCa ‘gating glutamate Glu203 or the ‘H^+^ glutamate site’ Glu270 to alanine prevented its activity in generating NO_3_^–^ flux-elicited currents or depolarization-induced H^+^ transport in oocytes (Bergsdorf *et al.*, 2009; Miller and Nguitragool, 2009), suggesting that the two Glu sites are H^+^-binding sites in AtCLCa.

## Cellular pH homeostasis during N assimilation

### The ‘proton economy’ in N transport and assimilation

**Fig. 3.**
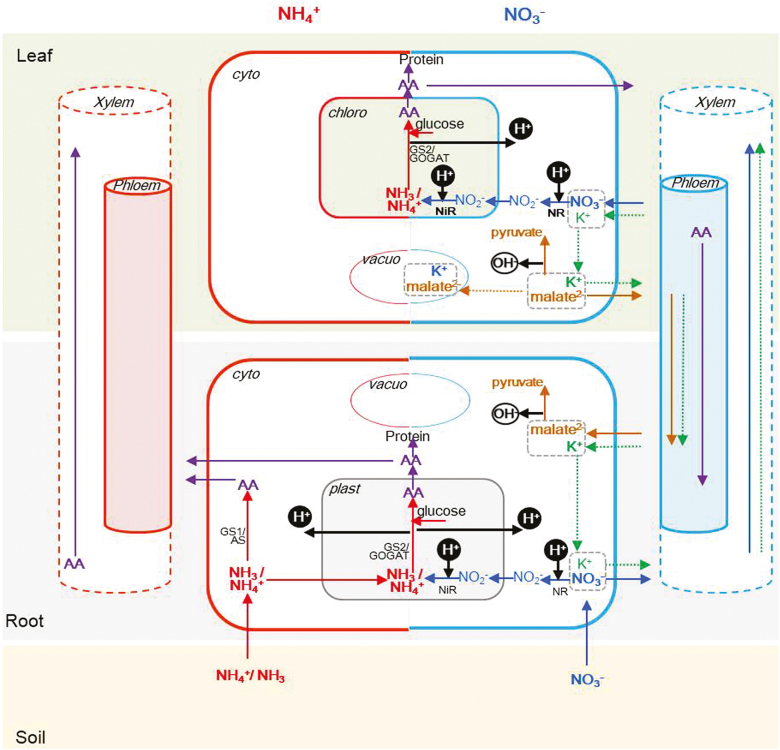
pH regulation during NH_4_^+^ and NO_3_^–^ assimilation. NH_4_^+^ transport and assimilation pathways are indicated by red arrows, NO_3_^–^ transport and assimilation by blue arrows, amino acid (AA) transport by purple arrows, malate transport and assimilation by brown arrows, K^+^ transport by green arrows, and H^+^ or OH^–^ production or consumption by black arrows. NH_4_^+^ is assimilated mainly in roots, and NO_3_^–^ is assimilated in both roots and shoots, which are dependent on plant species and N supply levels (Raven and Smith 1976; Andrews. 1986; Raven, 1986). The N assimilation requires ATP and carbon skeletons, glucose, malic acid (OAA), or malate. Malate accumulates in NO_3_^–^-supplied plants and can be stored in vacuoles, or transported to roots for further reactions (Raven and Smith, 1976). Malate converted to pyruvate helps overcome cytosolic acidification at low external pH (Raven and Smith, 1976). The assimilation of NH_4_^+^ produces at least one H^+^ per NH_4_^+^. The H^+^ produced are partially neutralized to counter the cytoplasmic akalinization caused by NH_4_^+^ transport in roots (Gerendás and Ratcliffe, 2000), or stored in vacuoles (Raven and Smith, 1976; Raven, 1986). NR, nitrate reductase. NiR, nitrite reductase. AS, asparagine synthetases. GS, glutamine synthetases. GOGAT, glutamine oxoglutarate aminotransferase. Cyto, cytosol. Vacuo, vacuole; chloro, chloroplast; plast, plastid.

The majority of root acquired NH_4_^+^ is rapidly assimilated in roots, whereas NO_3_^–^ is mainly assimilated in shoots depending on different plant species and the external N level, requiring both ATP and carbon (C) skeletons (Fig. 3; Table 2; Raven and Smith, 1976; Andrews, 1986; Bloom *et al.*, 1992; Rachmilevitch *et al.*, 2004; Nunes-Nesi *et al.*, 2010; Britto and Kronzucker, 2005).

**Table 2.** Proton changes in the processes of N transport and assimilation

N utilization processes		Equation of H^+^ change in cytoplasm
NH_4_^+^	NH_4_^+^ transport	NH_4_^+^_(out)→_NH_3_+H^+^_(out)_
	NH_3_ protonation	NH_3_+1H^+^_→_NH_4_^+^
	NH_4_^+^ assimilation	NH_4_^+^+C_6_H_12_O_6_+1.5O_2→_C_5_H_8_NO_4_^–^+CO_2_+3H_2_O+2H+
NO_3_^–^	NO_3_^–^ transport	NO_3_^–^_(out)_+H^+^_(out)→_ NO_3_^–^+1H+
	NO_3_^–^ reduction	NO_3_^–^+2/3C_6_H_12_O_6_+ 2O_2_+2H^+^_→_ NH_4_^+^+4CO_2_+3H_2_O
	NH_4_^+^ assimilation	NH_4_^+^+C_6_H_12_O_6_+1.5O_2→_C_5_H_8_NO_4_^–^+CO_2_+3H_2_O+2H+

H^+^, H^+^ production and H^+^, H^+^ consumption in the cytoplasm. In the process of NH_4_^+^ transport, it is assumed that 1NH_4_^+^ counterbalances 1 extra H^+^, released to outside the cell (out). In the process of NH_4_^+^ assimilation, if the glucose is ample, 2H^+^ will be produced in the cytoplasm. For 1NO_3_^–^/2H^+^ co-transport into the cytoplasm, it is assumed that 1H^+^ is pumped out of the cell by the PM H^+^-ATPase. For NO_3_^–^ reduction, 2H^+^ will be produced when plenty of carbon is available. Combining the NO_3_^–^ transport, reduction, and assimilation, if 1NO_3_^–^ is totally incorporated into 1 glutamate (Glu), it yields 1H^+^ in the cell, and 1H^+^ extra (Britto and Kronzucker, 2005). If 1NH_4_^+^ is transported and assimilated to 1Glu, it generates 1H^+^ in the cell, and 1H^+^ extra (Britto and Kronzucker, 2005).

Reduction of NO_3_^–^ to NH_4_^+^ is catalysed by nitrate reductase (NR) and nitrite reductases (NiRs) in the cytosol and plastids or chloroplasts, respectively, with the consumption of 2H^+^ (molecule) per 1NO_3_^–^ in the cytosol (Fig. 3; Table 2; Lea and Miflin, 1974; Xu *et al.*, 2012). In general, NH_4_^+^ assimilation into amino acids occurs quickly under NH_4_^+^ supply and is conducted in root plastids or shoot chloroplasts by the GS/GOGAT cycle, producing 2H^+^ per 1NH_4_^+^ (Fig. 3; Table 2; Masclaux-Daubresse *et al.*, 2010). However, there is some controversy as to whether the GS/GOGAT pathway of NH_4_^+^ assimilation is net H^+^ consuming or producing in plants. Three conditions need to be considered for predicting the consumption or production of H^+^ in NH_4_^+^ assimilation. (i) If ATP and NAD(P)H for the reaction are regenerated only by other processes, the GS/GOGAT pathway is H^+^ consuming (Kosegarten *et al.*, 1997). (ii) If the C skeletons can be continually provided for the regeneration of ATP and NAPD(P)H, it appears to be an H^+^-releasing process (Gerendás and Ratcliffe, 2000). (iii) If the C skeletons are limited, then the 2-oxoglutarate pool is replenished by re-utilization of malate (stored in the vacuole), and NH_4_^+^ assimilation may rapidly consume H^+^ (Gerendás and Ratcliffe, 2000). In addition, different plant species showed diverse cytoplasmic pH changes in response to NH_4_^+^; for example, rice, which has stronger GS activity than maize, showed a larger increase of cellular pH during NH_4_^+^ assimilation (Magalhaes and Huber, 1989, 1991; Kosegarten *et al.*, 1997).

The combination of NH_4_^+^ or NO_3_^–^ transport and assimilation results in different net changes of H^+^ numbers in plant cells (Table 2; Bloom *et al.*, 1992; Rachmilevitch *et al.*, 2004; Britto and Kronzucker, 2005). Incorporation of one NH_4_^+^ to glutamate produces one H^+^ in the cell, while assimilation of one NO_3_^–^ to glutamate produces one H^+^. However, when NO_3_^–^ or NH_4_^+^ is not immediately assimilated and presumed to accumulate, it is expected that the uptake of NO_3_^–^ is a transient cytosol-acidifying process whereas that of NH_4_^+^ is a transient cytosol-alkalinizing process.

### Biochemical malate pH-stat due to NO_3_^–^ assimilation

In the process of NO_3_^–^ reduction to NH_4_^+^, a substantial amount of the dicarboxylate malate can accumulate in the cytosol due to the anion deficit (van Beusichem *et al.*, 1988; Lüttge *et al.*, 2000; Pasqualini *et al.*, 2001). Cellular malate synthesis and degradation is important for regulation of the cytosolic pH (Smith and Raven, 1979; Hurth *et al.*, 2005). For example, knockout of the tonoplast malate transporter AttDT reduced the capacity of the mutant plant to overcome cytosolic acidification in leaf protoplasts (Hurth *et al.*, 2005). However, these mutants did not have a strong phenotype, but the effect of changing N supply form was not tested.

### pH regulation during amino acid transport

In addition to inorganic N, amino acids in the soil solution can also be directly taken up by roots (Tegeder and Masclaux-Daubresse, 2018). Inside the plant, amino acids are the major form of N for transport and re-distribution, particularly in NH_4_^+^-supplied plants (Tegeder and Hammes, 2018).

Most amino acid transporters function with a H^+^ co-transport mechanism and this has been shown for a broad range of amino acids, including neutral, cationic, and anionic amino acids (Tegeder and Masclaux-Daubresse, 2018; Tegeder and Hammes, 2018). The plant amino acid transporters show characteristic pH dependence in oocytes (Boorer *et al.*, 1996; Boorer and Fischer, 1997; Hirner et al., 1998, 2006; Fischer *et al.*, 2002). Although there is no evidence for their direct involvement in pH regulation, the root amino acid transporters can lead to a slight increase in rhizosphere pH (Näsholm *et al.*, 2009). In sterile conditions, amino acids can be used as a positive control for experiments comparing NO_3_^–^ and NH_4_^+^ as N sources.

## Future perspectives

### Developing the techniques to instantly monitor in real-time the dynamic changes of cellular pH by either N transport or H^+^ pumps in plants

For a better understanding of the underlying mechanisms of cellular pH homeostasis during N uptake and assimilation, it is essential to develop more molecular tools enabling *in vivo* measurements of pH in different intracellular compartments. Changes in proton concentrations are associated with both the N transporter and H^+^-pumping activity of ATPase (De Angeli *et al.*, 2009; Bassil *et al.* 2011; Shen *et al.*, 2013), thus both factors should be taken into account for pH regulation in plant cells. In tobacco, Martinière *et al.* (2013a) used a pHluorin-based pH sensor to directly measure pH of the endomembrane system, and found that luminal pH homeostasis in the *trans*-Golgi Network (TGN) and pre-vacuolar compartment (PVC) involved both V-ATPase-dependent acidification and H^+^ efflux mediated by the activity of the Arabidopsis Na^+^(K^+^)/H^+^ exchanger NHX5. In Arabidopsis protoplasts, Shen *et al.* (2013) used a modified pHluorin targeted to different organellar compartments for visualization and quantification of pH *in vivo.* Other pH sensors are also available for measurement of intracellular pH in plants (Martinière *et al.*, 2013b). Some H^+^-coupled NO_3_^–^ transporters (e.g. AtCLCa) and NH_4_^+^ transporters (e.g. PvAMT1;1) have also been identified as transporters leading to cytosolic pH changes in the oocyte system (Bergsdorf *et al.*, 2009; Ortiz-Ramirez *et al.*, 2011). However, there is still a lack of information about direct measurement of intercellular pH in nitrate transporter mutant plants. With the available tools for in *vivo* pH measurement using pH sensors (Shen *et al.*, 2013; Reguera *et al.*, 2015), it will now be possible to determine how they affect pH in the cytosol and endomembranes in the future.

### Role of N-controlled cellular pH homeostasis in enhancing abiotic stress tolerance

In acidic media, H^+^ and NO_3_^–^ excretion are tightly coupled. AtANPF2.7/NAXT1 mediated root NO_3_^–^ excretion, and PM-ATPase stimulated H^+^ excretion (Segonzac *et al.*, 2007). H^+^ stress enhanced NO_3_^–^ uptake mediated by NRT1.1 in Arabidopsis and caused significant rhizosphere alkalinization (Fang *et al.*, 2016), and thus decreased some heavy metal toxicity such as that of Cd and Pb (Mao *et al.*, 2014; Zhu *et al.*, 2019). It would be interesting to examine how much such N-controlled cellular pH homeostasis and effects on rhizosphere pH can regulate plant tolerance to other abiotic stresses, such as heavy metals, drought, or flooding and salinity.

### Using natural genetic variation or point mutation of key H^+^-binding residues in N transporters to enhance the cellular pH homeostasis within plants for improving N uptake and utilization

It is known that intracellular pH can be a signal for modulating downstream responses (Roos, 2000; Felle, 2001; Kader and Lindberg, 2010). In rice, overexpression of OsNRT2.3b, a cellular pH-sensing nitrate transporter, could buffer N transport-induced phloem alkalinization, and thus improve NUE, phosphate and iron mobilization, C metabolism, and grain yield (Fan *et al.*, 2016). This provides an exciting example for the possibility of utilizing pH-sensing transporters to improve plant NUE and growth. It is worth checking in other N transporters if there is a tight link between H^+^-binding residues and N transport activity at different medium pH. In future, utilizing the natural genetic variation among germplasm collections or making point mutations by gene-editing techniques of pH-sensing transporters may be a pathway for enhancing crop production at varied N supply levels and improving NUE.

### Revealing the molecular regulatory mechanisms of synergism, antagonism, and interaction of NO_3_^–^ and NH_4_^+^ on potassium and other nutrients

In plants, the transport and assimilation of NO_3_^–^ and NH_4_^+^ can dominate cellular pH homeostasis, which in turn affects the availability and utilization of other nutrients. The synergism, antagonism, and interaction among N and other major nutrients, such as K^+^, Ca^2+^, Mg^2+^, and Cl^–^, are known to be physiologically relevant, while the regulatory mechanisms linking these nutrients to cellular pH homeostasis are unclear. Inactivation of some nitrate transporters, such as AtNPF7.3/NRT1.5 (Lin *et al.*, 2008; Drechsler *et al.*, 2015; Li *et al.*, 2017) and OsNPF2.4 (Xia *et al.*, 2015), affects both NO_3_^–^ and K^+^ distribution, showing that K^+^/NO_3_^–^ transport is tightly coordinated. However, more thorough investigation of the interactions between N and other nutrients are needed.
